# Supplementary material for: Exploring Genetic Determinants: A Comprehensive Analysis of Serpin B Family SNPs and Prognosis in Glioblastoma Multiforme Patients
Source: Cancers (Basel). 2024 Mar 10;16(6):1112. doi: 10.3390/cancers16061112 (PMC10968849; doi:10.3390/cancers16061112)
Supplement: Supplementary file 1 [file cancers-16-01112-s001.zip › cancers-2871116-supplementary.pdf]

TITLE OF RESEARCH STUDY:

Exploring Genetic Determinants: A Comprehensive Analysis of SerpinB Family SNPs and Prognosis in Glioblastoma Multiforme Patients

Please answer the following questions by ticking the response that applies

1. I have read the Information Sheet for this study and have had details of the study explained to me.

YES ☐ **NO** ☐

2. My questions about the study have been answered to my satisfaction and I understand that I may ask further questions at any point.

YES ☐ **NO** ☐

3. I understand that I am free to withdraw from the study within the time limits outlined in the Information Sheet, without giving a reason for my withdrawal or to decline to answer any particular questions in the study without any consequences to my future treatment by the researcher.

YES ☐ **NO** ☐

4. I agree to provide information to the researchers under the conditions of confidentiality set out in the Information Sheet.

YES ☐ **NO** ☐

5. I wish to participate in the study under the conditions set out in the Information Sheet.

YES ☐ **NO** ☐

6. I consent to the information collected for the purposes of this research study, once anonymized (so that I cannot be identified), to be used for any other research purposes.

YES ☐ **NO** ☐

Participant's Signature: ____
Date: ___________

Participant's Name (Printed): ___________________________________

Contact details: ____

Researcher's Name (Printed): Sohaib M. Al-Khatib

Researcher's Signature: ______________________________________

Researcher's contact details:

Sohaib M. Al-Khatib, M.D.

Associate Professor

Department of Pathology and Laboratory Medicine.

Jordan University of Science and Technology

E-mail: smkhatib4@just.edu.jo

Please keep your copy of the consent form and the information sheet together.
